# Supplementary material for: The interactome of intact mitochondria by cross-linking mass spectrometry provides evidence for coexisting respiratory supercomplexes
Source: Mol Cell Proteomics. 2017 Dec 8;17(2):216–32. doi: 10.1074/mcp.RA117.000470 (PMC5795388; doi:10.1074/mcp.RA117.000470)
Supplement: Supplemental Data [file supp_RA117.000470_133922_0_supp_23978_fzffwf.zip › spectra_annotation/mito_spectra_annotation/4-1-2-1-5-1.pdf]

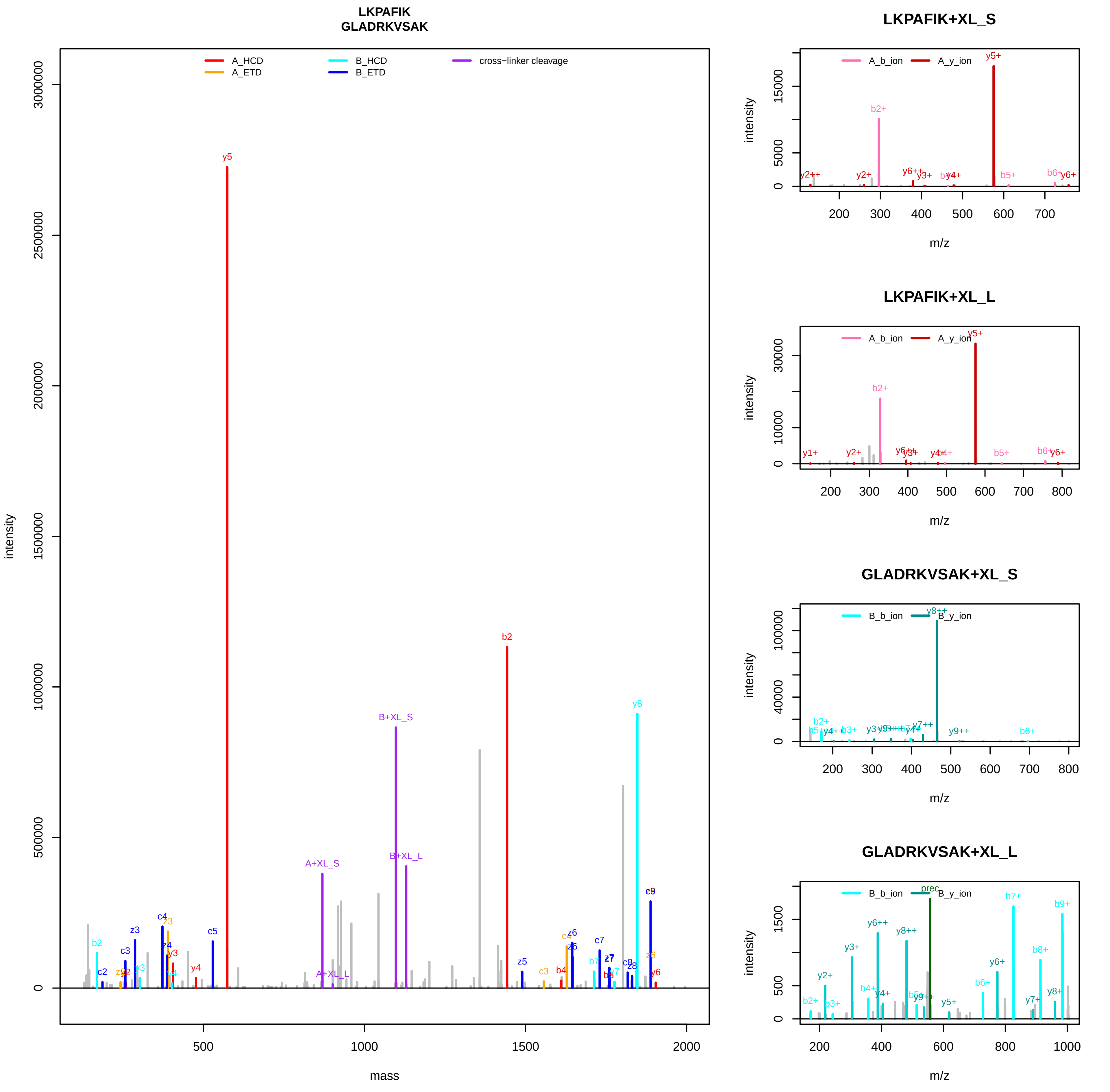
LKPAFIK
GLADRKVSAK
A_HCD
A_ETD
B_HCD
B_ETD
cross−linker cleavage
y5
b2
y8
B+XL_S
B+XL_L
A+XL_S
A+XL_L
c9
intensity
mass
0
500000
1000000
1500000
2000000
2500000
3000000
500
1000
1500
2000
LKPAFIK+XL_S
A_b_ion
A_y_ion
y5+
b2+
y2++
y2+
y6++
y3+
b5+
b6+
y6+
m/z
LKPAFIK+XL_L
y1+
GLADRKVSAK+XL_S
B_b_ion
B_y_ion
y8++
y9++
b6+
GLADRKVSAK+XL_L
prec
b7+
b9+
y6++
y8++
b8+
y6+
y7+
y8+
